# Home-Based Exercise and Fall Prevention in Older Adults: Development, Validation and Usability of the Mais Equilíbrio Mobile App

**DOI:** 10.2196/80724

**Published:** 2025-11-04

**Authors:** Mateus Medeiros Leite, Alessandro de Oliveira Silva, Silvana Schwerz Funghetto, Luciano Ramos de Lima, Samuel Barbosa Mezavila Abdelmur, Hudson Azevedo Pinheiro, Calliandra Maria de Souza Silva, Maurílio Tiradentes Dutra, Marina Morato Stival

**Affiliations:** 1 Physical Education Department, University Center of Brasilia - UniCEUB, University Center of Brasília, SEPN 707/907 - Asa Norte, Brasília - DF, 70790-075, Brasília, 70790-075, Brazil; 2 Graduate Program in Health Sciences and Technologies, Faculty of Ceilândia, Universidade de Brasília, Ceilândia Sul Campus Universitário - Centro Metropolitano, Brasília, 72220-275, Brazil, 55 61998541966; 3 Nursing Course, Faculty of Ceilândia, Universidade de Brasília, Brasília, Brazil; 4 Health Department of the Federal District, Governo do Distrito Federal, Brasília, Brazil; 5 Federal Institute of Education, Science and Technology of Brasília, Instituto Federal de Educação, Ciência e Tecnologia de Brasília, Brasília, Brazil

**Keywords:** mobile apps, elderly, physical exercise, fall prevention, mHealth interventions, older adults

## Abstract

**Background:**

The global aging population and the high incidence of falls among this population highlight the need for effective preventive strategies. Home-based exercise programs, such as the Otago protocol, have demonstrated efficacy in reducing fall risk but often face barriers related to user adherence. Mobile health (mHealth) apps offer promising tools to support health promotion and enhance autonomy in older adults.

**Objective:**

This study aims to develop and validate a prototype mobile app, *Mais Equilíbrio* (More Balance), designed to guide older adults in performing home-based physical exercises adapted from the Otago protocol.

**Methods:**

This methodological study was conducted in two phases: (1) content validation by 22 experts in physical education and physiotherapy using the Suitability Assessment of Materials (SAM) scale, and (2) usability testing with 24 older adults (aged 60 to 80 y), using the System Usability Scale (SUS). An overall score above 70% on the SAM and above 85 on the SUS were considered indicators of high quality and excellent usability, respectively.

**Results:**

The *Mais Equilíbrio* (More Balance) app was developed based on the Otago protocol and tailored for independent home use. A Content Validity Index above 0.95 was observed for all items. An overall average score of 81.20 (SD 15.78) on the SAM scale was found, classifying the material as “superior.” Usability tests with older adults showed an average score of 95.98 (SD 5.58) on the SUS, indicating excellent usability. The highest scores were observed in “ease of use” and “user confidence.”

**Conclusions:**

The *Mais Equilíbrio* (More Balance) app, distinct for digitally adapting the Otago protocol to the Brazilian context and for its dual validation process with experts and older adults, has proven to be a valid and highly usable tool for guiding home-based physical exercise in older adults, with potential to promote fall prevention and autonomy.

## Introduction

Population aging has accelerated in recent decades, driven by social, health, and technological advances that have increased life expectancy while reducing mortality and fertility rates [1]. Estimates indicate that this trend will persist, with significant growth in the older adult population expected over the coming decades, especially in high-income countries [2,3].

This scenario is accompanied by a higher prevalence of chronic diseases and aging-related conditions, such as falls. Approximately 28%‐35% of older adults experience at least one fall per year, and 15% report recurrent episodes. Falls contribute to nearly 300,000 deaths worldwide and are associated with severe consequences [4-6], including fractures, functional decline, fear of falling, and postfall syndrome, which often lead to loss of independence, reduced life expectancy, and increased morbidity and mortality [7-11].

Falls among older adults are multifactorial events influenced by intrinsic, extrinsic, and situational factors, including biological, behavioral, environmental, socioeconomic, psychosocial, and demographic aspects [7,12,13]. Specific risk factors such as advanced age, female sex, polypharmacy, history of falls, low cognitive performance [14], reduced physical function [15], decreased muscle strength (MS), particularly in the lower limbs [16], fear of falling, balance impairments, and low physical activity levels [17-21] further increase the likelihood of falls [22].

Low physical activity levels negatively affect older adults’ ability to perform activities of daily living and are associated with adverse health outcomes and higher mortality [23,24]. Consequently, interventions targeting MS and balance are recommended for fall prevention. Programs such as the Otago Exercise Program (OEP) and the Home-based Older People’s Exercise have demonstrated effectiveness, including in home-based implementations [25-27].

In this sense, mobile health (mHealth) technologies, an important component of gerontechnology, offer promising tools to support self-care, guide physical exercise practices, and assist in the monitoring of chronic health conditions [28-30]. In older adults, previous studies have demonstrated the use of mHealth solutions, especially with interventions that address health promotion, disease prevention, and long-term maintenance of regular exercise. Thus, these technologies stand out for their ability to be used independently and within home environments. However, the main barriers associated with this type of technology for older adults are declining motor and cognitive functioning, as well as low confidence in digital competence [31-34].

The development of the *Mais Equilíbrio* (More Balance) app was based on the Systematic Instructional Design model, which provides a structured framework for designing health-related apps [35]. To address the specific needs of the older population, the intervention design incorporated principles of universal usability and inclusive design. The choice of the OEP protocol as the scientific basis was motivated by its proven effectiveness in preventing falls, with risk reductions in older adults [4,36]. The central hypothesis is that technological mediation can overcome traditional barriers to adherence to home-based exercises, such as forgetfulness, low motivation, and lack of professional supervision.

Although several apps for fall prevention or risk assessment are already available, such as *FallSA*, designed as a self-assessment tool for community-dwelling older adults [37], the *Mais Equilíbrio* (More Balance) is distinctive in digitally integrating the OEP, adapted to the Brazilian context, with a strong focus on adherence and usability among older adults. Importantly, it also underwent a dual validation process, engaging both health experts and end users, which strengthens its contextual adequacy and reliability for future interventions. By converting the OEP into a mobile interface, *Mais Equilíbrio* (More Balance) combines the well-documented benefits of OEP, improvements in strength, balance, and fall reduction with the added advantages of digital health solutions, such as accessibility, remote monitoring, and scalability, benefits also highlighted in recent reviews of digitally delivered OEP interventions [38].

Despite the growing availability of mHealth solutions, there is a gap in the literature regarding the use of apps designed to promote physical exercise in older adults for improving balance, MS, and fall prevention. Most existing apps offer only educational content in text or images, with little interactivity, no self-monitoring, and no involvement of health professionals in the creation process. They also do not declare the scientific source of the content, which highlights the need for randomized controlled trials to assess their effectiveness. Given technological advancements, it is important to offer reliable material that uses appropriate language and attractive and dynamic features, and is easily accessible, facilitating understanding and encouraging home-based exercise routines [39].

Thus, this study aimed to develop and validate a prototype mobile app to promote home-based physical exercise in older adults, featuring instructional videos adapted from the Otago protocol.

## Methods

### Ethical Considerations

This study was conducted in accordance with national and international ethical guidelines and was approved by the Research Ethics Committee of the Faculty of Ceilândia, University of Brasília (Research Ethics Committee – CEP/FCE-UnB; approval number 5.530.239, approved on July 1, 2022). All procedures followed the guidelines of the National Research Council in accordance with current Brazilian legislation (Resolution No. 466/2012) and the principles of the Declaration of Helsinki. All participants provided informed consent prior to participation. The Free and Informed Consent Form (FICF) was obtained electronically through an online platform before data collection began. Participants were informed about the voluntary nature of the study and their right to withdraw at any time without penalty or loss of benefits. No financial or material compensation was provided to participants, in accordance with Brazilian ethical regulations for research involving human subjects. This research is part of the project entitled “Development and evaluation of home exercise technology for older adults".

### Study Design and Period

This is a methodological development study with a quantitative and descriptive approach, conducted from March 2023 to September 2024. The study followed the guidelines of the Revised Standards for Quality Improvement Reporting Excellence (SQUIRE 2.0) instrument.

### Implementation Scenario and Context

The development and validation of the *Mais Equilíbrio* (More Balance) app were carried out in the Brazilian health care system, which, despite a robust primary health care (PHC) model with extensive coverage, has limitations and still faces challenges in terms of individualized care to older adults. A strategic partnership with a basic health unit facilitated participant recruitment, optimizing access to the community. In this context, the following factors were considered crucial for the implementation of digital technologies: (1) availability of home internet infrastructure, (2) family support in the use of technology, (3) the socioeconomic heterogeneity of the population, and (4) public policies aimed at promoting the health of older adults.

### Technological Production: Prototype Development

To design the prototype of the *Mais Equilíbrio* (More Balance) mobile app, a structured workflow was established, with regular meetings between the development team and researchers to define the app’s interfaces and screens. The purpose of the app is to present video demonstrations of the adapted Otago exercise protocol, along with its progressive stages, as detailed in Table 1.

**Table 1.** Otago exercise protocol adapted for the *Mais Equilíbrio* (More Balance) app.^a^

	Warm-up	Strength	Balance	Walk
Activities	Neck movements Trunk rotation Trunk extension Ankle movements March	Knee extension Knee flexion Hip abduction Plantar flexion/Dorsiflexion	Squat Sitting down and standing up Tandem balance Balance 1 leg Walking underfoot Walking backwards Walk to the side Tandem walk Walking and turning Stair walking Walking on tiptoes	Encouraged
**Level**				
1 (2x week)	1× 10 repetitions	2× 10 to 12 repetitions	2× 10 to 12 repetitions	2x week 30 min
2 (3x week)	1× 10 repetitions	3× 10 to 12 repetitions	3× 10 to 12 repetitions	2x week 30 min
3 (3x week)	1× 10 repetitions	4× 10 to 12 repetitions	4× 10 to 12 repetitions	2x week 30 min

a Adapted from Campbell and Robertson [40].

The app was developed using a cross-platform framework to ensure compatibility across devices, with a backend designed to provide a secure and scalable architecture for data management. An administration panel was also implemented to allow efficient monitoring and management of the app. This combination of technologies enabled the creation of a responsive, user-friendly, and maintainable system suitable for long-term use [41].

The software was developed following the steps of Systematic Design of Instruction, a structured approach that systematizes the creation of mobile apps in the health care field. This model includes four phases: analysis, design or development, implementation, and evaluation [35]. In this stage, the analysis and design or development phases were carried out.

During the analysis phase, the software objectives were established by professionals with expertise in physical exercise for older adults, together with a digital interface designer and a programmer. The instructional content was developed using videos adapted according to the OEP protocol [26].

**Figure 1.**
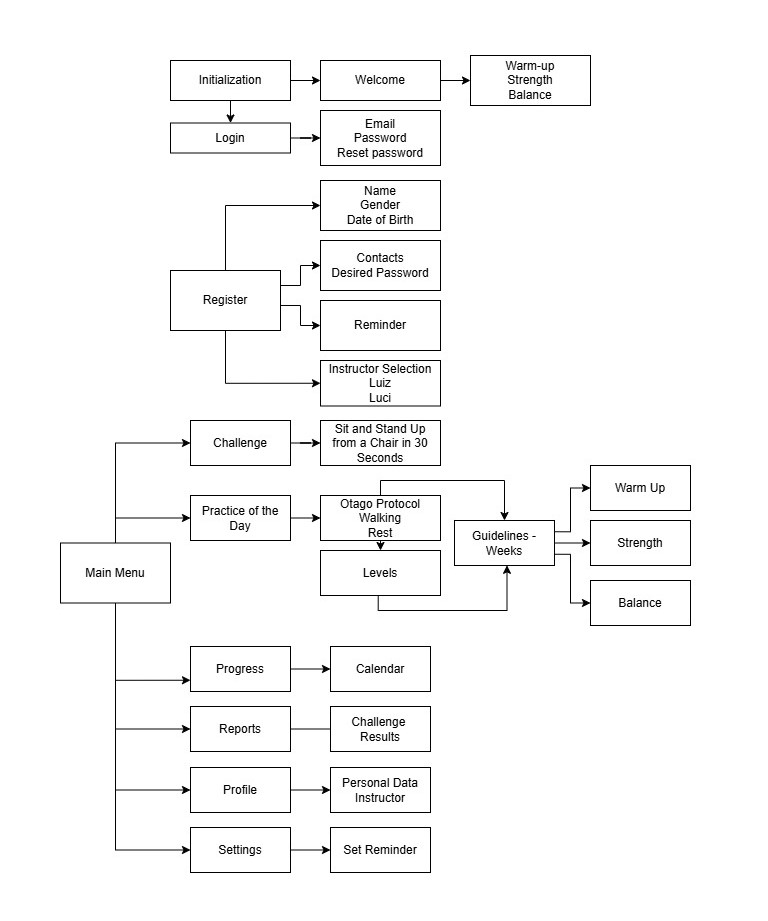
Framework of the *Mais Equilíbrio* (More Balance) app.

During the design or development phase, the researchers created videos tailored to language to enhance understanding among older adult users. The app’s layout composition was defined, ie, the visual organization and interaction models. For this activity, a digital interface designer and a programmer were consulted, both funded by the project. They developed the prototype for both Android and iOS operating systems, enabling future availability on the Play Store and Apple Store, thereby increasing the app’s reach and accessibility. The system framework was built in subdivisions of sections and subsections, as shown in Figure 1.

### Validation With Experts

A total of 310 experts were invited through different strategies, including dissemination on the Brazilian Lattes Platform of the National Council for Scientific and Technological Development, on institutional websites of major Brazilian universities, and through referrals from other experts who had already participated. The invitation letter was sent by email, along with the research objectives and a link to download the app, as well as access to the informed consent form and the app validation tool in Google Forms format. This instrument was structured into two sections: (1) characterization by experts and (2) Suitability Assessment of Materials (SAM) scale. The experts were selected using the method proposed by Fehring [42], with adaptations. A period of 30 days was granted for the completion of the validation, and, if it was not completed, a new email was sent every 7 days to reinforce the invitation.

The inclusion criteria for this group were as follows: being a physical education or physiotherapy professional; achieving a score ≥5 in Fehring’s adapted criteria (master’s degree: 4 points; master’s degree with dissertation in the area of interest: 1 point; research in the area of interest: 2 points; article published in the area of interest: 2 points; doctorate degree with thesis in the area of interest: 2 points; professional experience in the area of interest: 2 points; and specialization in the area of interest: 2 points); and agreeing to participate by fully completing all study-related forms. Experts were excluded from the study if they did not submit their assessment on time or if they only partially participated in the study. Ultimately, 22 experts agreed to participate in the research, meeting the previous recommendations [43].

For content validation, the SAM scale was organized on an ordinal scale that considered the following items: 0, inadequate; 1, adequate; and 2, excellent [44]. When selecting option 0, experts were asked to provide justification or suggestions for improvement. To calculate the overall SAM score, the arithmetic mean of the sums for each expert (Σ) was calculated, divided by the number of experts and multiplied by 100. This yielded the following percentages: 70%‐100% (superior material), 40%‐69% (adequate material), or 0%‐39% (inadequate material). A minimum score of 70% was required for the app to be considered approved [45].

### Validation With Target Audience

In this stage, older people aged 60 years or older (recognized as elderly in Brazil) who had access to smartphones with Android or iOS systems participated over the course of a week. The invitation to participate was made at a basic health unit providing PHC. Initially, 50 older adults were recruited. Of these, 24 were included, according to the inclusion criteria: age between 60 and 80 years; both sexes; literate; owning a smartphone that supports the app with internet access; walk without the aid of devices, as verified by self-report during screening. The usability of the app was evaluated in person using the System Usability Scale (SUS) [46,47].

To calculate usability by SUS, 1 was subtracted from the score of odd items, and for even items, the value of the 5-point scale was subtracted. The sum of the results was multiplied by 2.5, generating a final score between 0 and 100. Scores between 50 and 67 are considered borderline; 68‐84 indicate good usability; and above 85, excellent acceptance [48-50]. The assessment was carried out independently by two researchers, with joint review in case of discrepancies until consensus was reached. In addition to the overall score, the SUS items allow for the evaluation of aspects such as memorization (item 2), learning (items 3, 4, 7, and 10), efficiency (items 5, 6, and 8), satisfaction (items 1, 4, and 9), and inconsistencies (item 6) [51,52].

To ensure completeness and accuracy of the data collected, several control measures were employed. The evaluation forms (SAM, Content Validity Index [CVI], and SUS) were reviewed immediately after completion to ensure that all questions had been answered. For the usability validation with older adults, qualitative feedback was recorded through detailed notes to complement quantitative data and to ensure the reliability of the information. Quantitative data were transcribed and subjected to a double-checking stage by an independent researcher to identify and correct possible typing errors or inconsistencies. In addition, all instruments were applied in standardized environments, and researchers were previously trained to ensure uniformity in data collection procedures.

### Statistical Analysis

Data obtained were extracted from Google Forms to a Microsoft Excel (version 240) spreadsheet. IBM SPSS (Statistical Product and Service Solutions) software, version 25.0, was used for statistical analysis. The characteristics of the sample were categorized and presented by frequency and percentages. For content and appearance validation, the CVI was calculated to measure the experts’ agreement on the validity of the educational technology content [53-55]. To determine the level of agreement among experts, CVI scores were calculated using the number of experts who chose response options 1 or 2 for an item divided by the total number of experts who evaluated the item. The average CVI values for all messages and the content validity of individual items were calculated. A CVI greater than or equal to 0.78 was considered to indicate content validity [56,57].

The exact binomial distribution test indicated for small samples was performed, with a statistical significance level of *P* value <.05 and a concordance ratio of 0.95, to estimate the statistical reliability of the CVI. The internal consistency of the usability instrument (SUS) was verified by Cronbach α. The analysis of qualitative data, which included comments and suggestions from both experts in Stage 1 and participants in Stage 2, was performed using thematic content analysis. This approach allowed for an in-depth understanding of the aspects perceived as positive and the areas that required adjustments in the app, providing valuable insights into content and usability. Due to the cross-sectional design of this study, the evaluation of temporal effects on the variables was not applicable.

## Results

The development of the *Mais Equilíbrio* (More Balance) app was an iterative process, led by a multidisciplinary research team composed of specialists and professionals in care for older people, aligned with Systematic Instructional Design. This process resulted in several modifications, such as adjustments to the interface, simplification of commands, and inclusion of audio reminders, based on internal feedback from the team.

**Figure 2.**
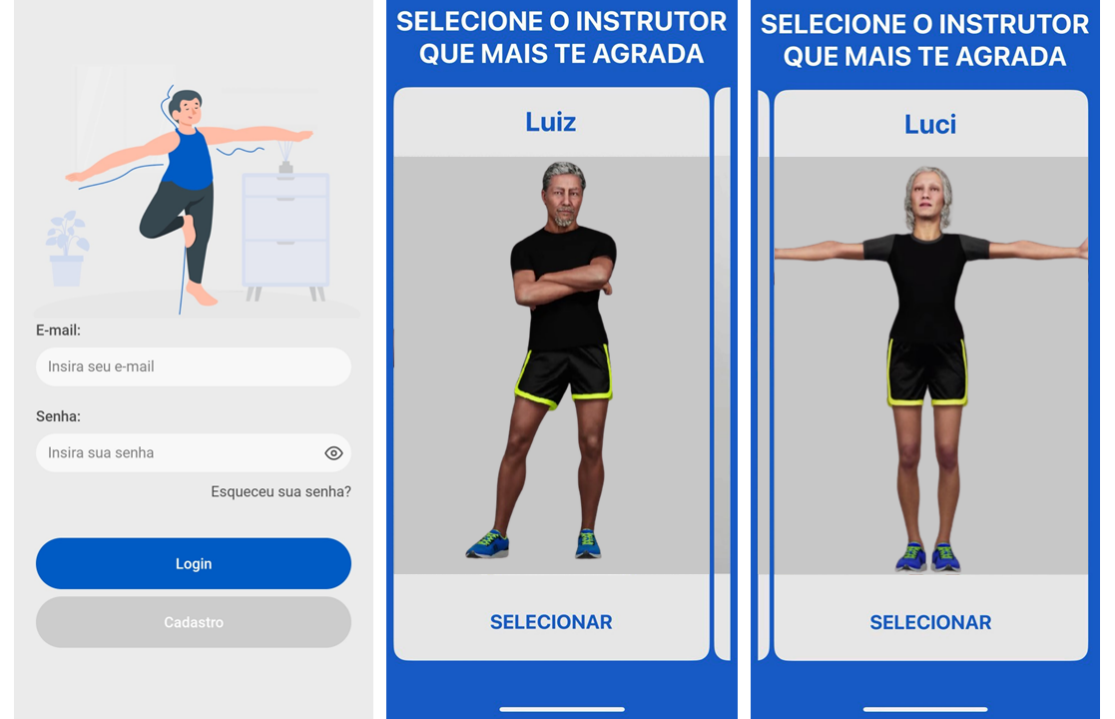
Tabs of the *Mais Equilíbrio* (More Balance) app. The figure shows three screens from the “*Mais Equilíbrio* (More Balance)” app. The first screen on the left displays a login interface, with fields labeled “E-mail” (“Enter your e-mail”), “Senha” (“password”), and buttons for “Login” and “*Cadastro*” (“Register”). The two screens on the right present the user with the option to select an instructor, with the title “SELECIONE O INSTRUCTOR QUE MAIS TE AGRADA” (“SELECT THE INSTRUCTOR YOU LIKE THE MOST).“ The two available instructors are Luiz and Luci, each depicted with their name above and a “*SELECIONAR*” (“SELECT”) button below.

Figure 2 presents an overview of the app’s interface and features through a collection of main screens. This visual representation includes everything from the authentication process to the monitoring and customization features, providing a complete overview of the user experience. To ensure fidelity to the study validation, the screens are presented in their original language, Portuguese, with specific details for each one provided in the figure caption.

Among the 22 experts who participated in the validation process, most were female (n=12, 54.55%), aged between 40 and 49 years (n=11, 50%), and physical education professionals (n=15, 68.18%). Regarding their qualifications, all held a master’s degree (n=22, 100%), and most also held a doctorate (n=18, 81.82%). In terms of scientific production related to digital technologies, fall prevention, balance, or MS in older adults, 54.55% (n=12) had a master’s dissertation on one of these topics, 45.45% (n=10) had a doctoral thesis in the area, and 86.36% (n=19) had published articles and ongoing research on the subject.

The results of the experts’ evaluation demonstrated the adequacy of the educational material, considering the criteria of content, language, presentation, methodology, and cultural relevance. All 22 items assessed obtained a CVI equal to or greater than 0.95. Twenty items achieved a CVI of 1.00, with total agreement among the evaluators. Three items obtained 95.45% agreement (CVI 0.96), remaining within the validation parameters (Table 2).

**Table 2.** Expert agreement on material suitability assessment items, Brasília, Federal District, Brazil, 2025 (n=22).

	Values	CVI^a^^,^^b^	*P* value^c^
Objective, n (%)		1.00	
The objective is clear, covers the proposed topic, and facilitates understanding of the material	22 (100)	1.00	.32
Content covers relevant information to meet the objective	22 (100)	1.00	.32
The material proposal is limited to the objectives, clarifying doubts about the addressed topic	22 (100)	1.00	.32
Content highlights key points and encourages behavioral change	22 (100)	1.00	.32
Language, n (%)		0.99	
Reading level is appropriate for targeted audience comprehension	22 (100)	1.00	.32
Language style facilitates understanding and allows for active engagement of the targeted audience	22 (100)	1.00	.32
Appropriate language using everyday words	21 (95.5)	0.96	.70
Context comes before new information	22 (100)	1.00	.32
Logical sequence of ideas and the learning process facilitated by items	22 (100)	1.00	.32
Graphic Illustrations, n (%)		1.00	
Design and graphic resources express the objective	22 (100)	1.00	.32
Types of illustrations and graphic resources	22 (100)	1.00	.32
Relevant graphic resources	22 (100)	1.00	.32
The graphic resources used are self-explanatory	22 (100)	1.00	.32
Resources have explanatory captions	22 (100)	1.00	.32
Layout and presentation, n (%)		0.97	
Layout appearance	22 (100)	1.00	.32
Dimension and quality of spelling	21 (95.5)	0.96	.70
Use of subtitles	21 (95.5)	0.96	.70
Motivation, n (%)		1.00	
There is interaction between the text or graphic resources and the targeted audience, leading them to make choices or demonstrate skills	22 (100)	1.00	.32
Desired behavior patterns that are well modeled or well demonstrated/exemplified	22 (100)	1.00	.32
There is motivation for self-efficacy, that is, people are motivated to learn because they believe that tasks and behaviors are feasible	22 (100)	1.00	.32
Cultural suitability		1.00	
The material is culturally appropriate to the logic, language, and experience of the targeted audience, n (%)	22 (100)	1.00	.32
Features culturally appropriate images and examples, n (%)	22 (100)	1.00	.32
SAM^d^ global score, mean (SD)	81.20 (15.78)	—^e^	—

a Item content validation index.

b CVI: Content Validity Index.

c Binomial test.

d SAM: Suitability Assessment of Materials.

e Not applicable.

The average SAM global score was 81.20 (SD 15.78) points, classifying the material as “superior” according to the instrument’s criteria. Thus, the results indicate that the content of the app was considered adequate in terms of clarity, relevance, accessible language, visual organization, and adaptation to the targeted audience (Table 2).

The target audience (older people) participated in the usability validation stage but were not involved in the development interactions. During this validation stage with the older people, it was observed that contextual elements, such as family support and internet access, interacted with the user experience. No direct adverse consequences were identified. However, based on the reports of the validation participants, unexpected benefits emerged, such as increased self-confidence in the use of technology and improvement in exercise routines. Rigorous data collection ensured that no relevant data were missing.

In the usability validation by 24 older adults, the majority were female (n=16, 66.67%); aged between 60 and 69 (n=18, 75.00%); had completed high school, undergraduate, or postgraduate degrees (n=18, 75.00%); and exercised (n=18, 83.33%). Table 3 shows means and SDs of the SUS domain scores by the target audience. The overall mean scale score was 95.98 (SD 5.58) points. Among the evaluated domains, the domain *Ease of memorization* obtained an average of 100.00 points, followed by the domains *System efficiency* and *Inconsistencies*, both with an average of 96.88 (SD 3.13 and SD 15.31 points, respectively). The domain *Ease of system knowledge* had an average of 94.79 (SD 2.55). Finally, the domain *User satisfaction* had an average of 94.44 (SD 3.18) points. The individual questions on the scale had averages ranging from 91.67 to 100.00 points.

**Table 3.** Distribution of average scores for the domains of the System Usability Scale for the *Mais Equilíbrio* (More Balance) app (n=24).

Usability feature	Scores, mean (SD)	Meaning
Ease of use	94.79 (2.55)	Easy-to-use system when used for the first time
Q3^a^	97.92 (7.06)	
Q4	91.67 (19.03)	
Q7	94.79 (12.72)	
Q10	94.79 (20.82)	
System efficiency	96.88 (3.13)	Speed in executing established tasks
Q5	93.75 (21.17)	
Q6	96.88 (15.31)	
Q8	100.00 (0.00)	
Inconsistencies	96.88 (15.31)	Absence of errors
Q6	96.88 (15.31)	
Ease of memorization	100.00 (0.00)	Easy to use even after a long period of not using it
Q2	100.00 (0.00)	
User satisfaction	94.44 (3.18)	Pleasant design
Q1	93.75 (21.17)	
Q4	91.67 (19.03)	
Q9	97.92 (7.06)	
SUS^c^ Global Score	95.98 (5.58)	—^b^

a Q: System Usability Scale question.

b Not applicable.

c SUS: System Usability Scale.

## Discussion

### Main Results

The *Mais Equilíbrio* (More Balance) app was developed to encourage and educate older adults to adopt self-care and fall prevention practices, thereby promoting health and quality of life. To this end, this study aimed to develop and validate this prototype mobile app for promoting home-based physical exercise, containing videos that demonstrate adapted exercises based on the Otago protocol.

Managing health in old age can be challenging. Older people are often less likely to trust their own ability to access health information and advice, especially online. This reluctance can diminish their interest in using digital health information and advice modalities [58]. To reverse this situation, it is important to optimize information processing and minimize cognitive load for effective health education. This means promoting access to the digital era and stimulating interest in online health information, especially through reliable sources and with health professionals’ participation. These strategies can contribute to autonomy, self-care, and health management during aging [59,60].

In this context, mobile technologies combined with internet access have great potential to contribute to health promotion and disease prevention, enabling access to knowledge and the development of skills and responsibilities necessary for the adoption of healthier behaviors [33,61]. The *Mais Equilíbrio* (More Balance) app can provide accessibility and usability for older people, focusing on simplicity, clarity, and the use of objective, audience-adapted language.

To facilitate use, buttons have been enlarged and strategically positioned. Content has been organized into blocks with intuitive navigation and voice assistance for the actions to be performed. To make the app more inclusive, visual elements considered common age-related visual limitations, employing large fonts, high-contrast colors, simple and recognizable icons, and clear, uniform illustrations. The content was built based on recommendations from guidelines and technical materials on elderly health, ensuring relevance and quality, prioritizing comprehensibility and usefulness [55,62-65].

It is believed that the user-centered approach was able to establish joint participation between the target audience and the design/research team, which is essential from the initial stages of conception [66]. In addition, innovative operational features were integrated to promote regular exercise adherence, such as customizable alarm reminders, which users can adjust according to their routines.

Unlike many digital health apps that do not undergo rigorous evaluation—leading to gaps in quality, accessibility, and coverage of user needs—this study included a structured validation process with expert participation [67,68]. The results obtained show high levels of agreement among the evaluators, which reflected in the satisfactory CVI values, confirming the validity and reliability of the app. Such validation is essential to ensure safety, efficacy, and accessibility of digital health tools for older adults [68-70].

Validation by the target audience allowed us to verify the usability and content of the app, according to principles of comprehensibility and suitability to the educational and cultural profile of the participants, highlighting ease of use, simplicity of the interface, and memorization of functions. In this research, we opted to use the SUS Scale, and studies show that an SUS score above 68 indicates an acceptable degree of usability, while scores above 85 would be associated with excellent acceptance of software or an app [46,71]. In the present study, an overall score of >90 points was obtained, confirming high usability, with no inconsistencies in the responses. This suggests strong potential for adherence, satisfaction, and effectiveness of the app as a tool to support physical activity among older adults.

In this perspective, other studies focusing on the use of technologies to track and prevent fall episodes have been developed [37]. The researchers developed FallSA, a mobile fall screening app, and determined its acceptance, concurrent validity, test–retest reliability, discriminatory ability, and predictive validity as a self-screening tool to identify fall risk among older adults in Malaysia. The results of that study indicated a high level of acceptance, with 80% of older adults agreeing with its suitability as a fall self-screening tool, with moderate to strong discriminatory ability in classifying older adults as fallers and non-fallers [37].

In recent years, additional studies have expanded the evidence base beyond FallSA. In a randomized controlled trial combining wearable activity trackers with a home-based multicomponent exercise program in older adults with a history of falls, significant improvements in fear of falling and physical function were observed after 12 weeks [72]. Other app-based interventions, which prescribed exercise using reinforcement learning, demonstrated greater user satisfaction and higher exercise intensity under adaptive conditions [73]. These findings highlight the importance of systematic design guidelines for mobile apps targeting older adults, emphasizing usability features such as simplified navigation and visual clarity [69]. Such principles align with the design choices implemented in the *Mais Equilíbrio* (More Balance) app, supporting the notion that its dual focus, adaptation of the OEP protocol, and usability validation are consistent with current best practices in digital fall-prevention interventions.

Unlike previous mHealth solutions for fall prevention, which often focus exclusively on self-assessment tools [37], or generic exercise guidance without a validated scientific protocol [69,72,73], the *Mais Equilíbrio* (More Balance) app provides a unique contribution by digitally adapting and delivering the evidence-based OEP in the Brazilian context. Its dual validation process with both health professionals and older adults ensures not only scientific rigor but also usability and acceptability in the target population, addressing key barriers to adherence in home-based exercise interventions.

As such, it is believed that this kind of validation, based primarily on updated technical guidelines and protocols, ensures safety, quality, and effectiveness in health care. This is in line with the fundamental principles of usability assessment, especially when used on mobile devices by older adults. These results corroborate other studies that highlighted the need for design adaptations and combined alternatives for solutions, aiming to improve older adults’ engagement with mHealth interventions [74]. Thus, usability testing is necessary before making the app available to the end user, as discussed in other studies [47,70,75].

Acceptance of usability by the target audience is directly associated with adherence to and continued use of health technologies [76]. In this sense, the validation carried out in this study revealed high scores in the SUS domains, highlighting the ease of use of the system from the first use, the agility in performing the proposed tasks, the low occurrence of errors, the ease of reuse even after a long period without contact with the system, in addition to a pleasant design. These results are consistent with findings from previous studies that identify key usability factors influencing user adherence, such as ease of use, efficiency through simple navigation, intuitive tasks, and accessible design [77-79]; high satisfaction and acceptance driven by a positive perception of the tool’s usefulness [80,81]; and facilitated learning, characterized by fewer barriers and increased confidence among older adults in using technology [82].

### Limitations

This study has some limitations. First, the validation was conducted over a limited time period, making it impossible to evaluate the prolonged use of the app or its effects on user behavior over time. Another limitation is related to the absence of clinical or functional assessment of participants regarding falls, which could deepen understanding of the practical impact of the tool. In addition, the absence of formal economic analyses, such as cost-effectiveness and budgetary impact, represents an important gap in assessing the sustainability and feasibility of large-scale implementation in the public health system. To this end, we aim to soon evaluate the prolonged use of the app or its effects on user behavior, adding clinical and functional assessments to measure the app’s effectiveness in reducing fall risk, improving balance and MS, as well as incorporating economic analyses to understand costs and benefits. Furthermore, cognitive status, an important factor in studies involving older adults, was not formally assessed, which should be considered when interpreting the findings. Most older adult participants were women (n=16; 66.7%), which may limit generalizability and highlight potential gender differences in usability perception. Finally, as this study focused only on usability validation, there is no evidence of clinical outcomes, such as improved balance or reduced falls, and this limitation should be acknowledged. Future studies with larger, more diverse samples, including cognitive, clinical, and functional assessments, are warranted to provide more robust evidence of the app’s effectiveness and sustainability.

### Clinical and Research Implications

The app has proven to be a promising tool for promoting the health of older adults, with the potential to support fall prevention through accessible educational content and guided exercises. Among its strengths are its validation by physical education and physical therapy experts, its intuitive interface, and its high level of usability. Future studies may test the app’s effectiveness with older adults in continuous use, evaluate health outcomes, and explore its effectiveness in reducing fear of falling, risk of falls, and increasing MS and balance through the exercises contained in the app.

In practical terms, integrating *Mais Equilíbrio* (More Balance) into Brazil’s PHC system could follow community-based mHealth models used in other low- and middle-income countries (LMIC). Mobile interventions have improved strength, balance, and self-efficacy in preventing falls among older adults [83], while reviews emphasize the need for user-friendly design, trained health professionals, infrastructure, and alignment with public health policies [84].

In Brazil, the involvement of community health workers and family health teams may enhance the adoption and dissemination of the app, while its integration into routine primary care activities could support scalability and long-term sustainability in fall-prevention initiatives. Beyond local implementation, *Mais Equilíbrio* (More Balance) also aligns with broader policy agendas. Both the Sustainable Development Goals and the World Health Organization Global Digital Health Strategy emphasize the development of innovation ecosystems to strengthen PHC and improve population health [85]. Consistently, the Brazilian *Digital Health Strategy 2020‐2028* establishes priorities for building an interconnected ecosystem that brings together the *Sistema Único de Saúde* (Unified Health System), technology companies, startups, universities, and research centers. A key action is to promote innovation environments that leverage digital connectivity to meet users’ needs [86], underscoring the potential of *Mais Equilíbrio* (More Balance) to be scaled within the SUS and adapted to other LMIC contexts.

### Conclusions

This study developed and validated the mobile app *Mais Equilíbrio* (More Balance), a digital tool designed to guide home-based physical exercises adapted for older adults. Content validation by experts ensured quality and instructional relevance, while high usability and acceptance confirmed by the older adults themselves attest to the tool’s applicability and comprehensibility. The results confirmed *Mais Equilíbrio* (More Balance) as an understandable and applicable tool ready for use.

Therefore, the findings reinforce the potential of mHealth technologies to overcome barriers to health access, especially in the context of PHC, where challenges such as limited access to professionals and resources persist in LMICs. The high acceptance of the app by the target audience reaffirms its relevance. To ensure the sustainability of the intervention and expand its dissemination, we propose integrating the app into PHC health promotion strategies, training community agents to provide technical support, and establishing partnerships with health departments and active aging programs. In addition, this study offers valuable contributions to the development and validation of health communication strategies through mobile apps, highlighting the importance of training older adults in self-management of health, especially in the context of physical activity and fall prevention.

Although *Mais Equilíbrio* (More Balance) represents a valuable contribution to increasing research on digital health and the validation of interactive tools, future studies are needed to assess its long-term clinical effectiveness and impact on objective health outcomes in the older adults population, especially in fall prevention. It is also essential to explore the adaptation of the app to different cultural and linguistic contexts and to investigate the economic feasibility and financial sustainability of its large-scale implementation.

## Abbreviations

CVI: Content Validity Index
LMIC: low- and middle-income countries
mHealth: mobile health
MS: muscle strength
OEP: Otago Exercise Program
PHC: primary health care
Q: Question (related to System Usability Scale)
SAM: Suitability Assessment of Materials
SQUIRE 2.0: Revised Standards for Quality Improvement Reporting Excellence
SUS: System Usability Scale
